# Enhancing sustainability competencies: A systematic review of educational interventions for dietitians and nutrition professionals

**DOI:** 10.1111/1747-0080.70046

**Published:** 2025-09-17

**Authors:** Romina Lörzing, Tina Bartelmeß

**Affiliations:** ^1^ Institute for Management in Medicine and Health Sciences, Faculty of Law, Business and Economic University of Bayreuth Bayreuth Germany; ^2^ Junior Professorship of Food Sociology, Faculty of Life Sciences: Food, Nutrition and Health, Campus Kulmbach University of Bayreuth Kulmbach Germany

**Keywords:** education, nutritionists, professional competence, sustainable development goals

## Abstract

**Aims:**

This study investigates how sustainability competencies are integrated into educational interventions for dietitians and nutrition professionals and examines pedagogical approaches employed, offering insight into how well these professionals are prepared to support the transition towards a sustainable food system.

**Methods:**

A systematic review was conducted using four electronic databases (PubMed, SCOPUS, Web of Science and ProQuest) and additional manual searches on 18 June 2024. Studies were included if they focused on educational interventions for dietitians and nutrition professionals that addressed sustainability competencies. Study quality was appraised using Johanna Briggs Institute critical appraisal tools and the Mixed Methods Appraisal Tool. Results were narratively synthesised and tabulated.

**Results:**

Nineteen studies met the inclusion criteria. While certain sustainability competencies are addressed, competencies related to inter‐personal, integration, intra‐personal and futures‐thinking remain underrepresented. Practical approaches with high potential for fostering sustainability competencies, such as project‐/problem‐based learning and place‐based environmental education, were frequently implemented. These methods were often combined with less effective instructional strategies, such as traditional lecturing.

**Conclusions:**

Education for dietitians and nutrition professionals should adopt a more comprehensive approach, integrating all sustainability competencies across the curriculum. Combining practical, experiential methods with traditional lecturing may enhance sustainability learning.

## 1 INTRODUCTION

Our current food system is both unhealthy and unsustainable, contributing not only to climate change, deforestation and biodiversity loss, but also to the global rise of chronic diseases such as obesity, diabetes mellitus and cardiovascular diseases.^1^ The concept of Planetary Health emphasises that human health and civilisation are linked to the integrity of Earth's natural systems. Human activities that disrupt these systems ultimately threaten human wellbeing.^2^ Consequently, a transformation of our food system is essential to promote both a healthy planet and human wellbeing.^3^, ^4^ Dietitians and nutrition professionals play a key role in transforming the food system,^5^ as they work in different practice areas and contexts in which they can implement sustainable practices, for example, by promoting sustainable food procurement policies or strategies to reduce food waste. They can also support individuals to raise awareness of the environmental impact of eating habits and advice about sustainable diets.^6^, ^7^, ^8^ Therefore, the training of dietitians and nutrition professionals plays a crucial role in equipping them with necessary skills and knowledge to understand how they can contribute to food system transformation.^5^ In this paper, sustainability is defined following the United Nations' concept of ‘meeting the needs of the present without compromising the ability of future generations to meet their own needs.’^9^ In the context of food systems, we refer to the Food and Agriculture Organisation's definition of a sustainable food system as one that ensures food security and nutrition for all, while maintaining the economic, social and environmental foundations necessary to provide food for future generations.^10^

However, many dietitians and nutrition professionals lack the education needed to apply these sustainable practices.^11^ To integrate sustainability effectively into their work, they require structured, holistic training.^11^ A survey of European dietitians highlights their willingness to learn and be trained about sustainability.^12^ Previous research has indicated that sustainability is already incorporated into the education of dietitians and nutrition professionals, albeit inconsistently and to varying degrees. Content on sustainable food systems is not systematically integrated into all training standards, and learning outcomes are often defined at a low or variable level of cognitive complexity.^13^, ^14^ A recent study revealed a positive trend of increased integration of planetary health content into nutrition and dietetics curricula. However, the study also highlighted that learning outcomes were primarily at a lower level of cognitive complexity, indicating room for improvement.^15^ Furthermore, diverse pedagogical approaches and evaluation methods are employed when teaching sustainability,^16^, ^17^ creating challenges to educators in selecting suitable teaching strategies.^16^ However, previous research has not thoroughly analysed which sustainability competencies are explicitly addressed or how educational interventions are structured to develop these sustainability competencies.

Therefore, this review aims to analyse educational interventions for dietitians and nutrition professionals with regard to the consideration of relevant competencies for advancing sustainability.

The following research questions will be answered:

Research question 1: To what extent do educational interventions for dietitians and nutrition professionals address sustainability competencies, enabling them to support system change for sustainable food systems?

Research question 2: What pedagogical approaches have been used in these educational interventions?

In this review, the term education refers to a wide range of learning formats, including formal secondary and tertiary education, such as vocational training and university programs, as well as non‐formal educational activities. The in‐depth analysis of educational interventions is based on two theoretical frameworks. The unified framework of competencies for advancing sustainability transformations by Redman and Wiek^18^ was selected for its comprehensive definition of sustainability competence. This framework identifies eight key competencies—systems‐thinking, futures‐thinking, values‐thinking, strategies‐thinking, inter‐ and intra‐personal competencies, implementation competence and integration competence—complemented by disciplinary, general and professional competencies.^18^ It provides a basis for assessing how and which areas of sustainability competencies are addressed in the educational interventions (research question 1).

Moreover, the effective development of these competencies depends on appropriate pedagogical approaches.^19^, ^20^ Lozano et al.^21^ propose a framework that illustrates the relationship between pedagogical approaches and the development of sustainability competencies. This framework serves as a structured guide for educators in selecting pedagogical approaches that can be used to specifically develop and promote sustainability‐related competencies in the classroom.^21^ In this study, it enables a systematic analysis of the pedagogical approaches used in educational interventions and their alignments with targeted sustainability competencies (research question 2).

## 2 METHODS

This systematic review is reported according to the Preferred Reporting Items for Systematic Reviews and Meta‐Analysis^22^ guidelines and was prospectively registered on the Open Science Framework (osf.io/bqzu2). Eligibility criteria were defined using the population, intervention, comparator and outcomes framework as follows (see Table 1). Studies were included if they focused on dietitians, nutritionists or similar professionals in related fields (e.g., students from nutrition‐related study programs like Human Nutrition or dietetic interns) and involved an educational intervention of any type. No restrictions were applied regarding the level or form of education. Interventions at the secondary and tertiary levels, as well as from both formal and non‐formal educational contexts, were included. Studies without educational interventions or those focused solely on non‐nutrition professionals were excluded. No restrictions were placed on comparators. Eligible studies had to address at least one sustainability‐related learning outcome as defined by Redman and Wiek.^18^ All empirical designs, including experimental, quasi‐experimental, observational, qualitative, quantitative and mixed methods, were considered. Furthermore, only articles published in German or English were included due to the language capabilities of the researchers.

**TABLE 1:** Eligibility criteria based on the Population Intervention Comparator Outcome framework.

	Inclusion	Exclusion
Population	Dietitians, nutrition professionals or similar professions (e.g., students of nutrition‐related study programs)	No dietitians, no nutrition professionals; professions not related to nutrition, medical students
Intervention	Educational intervention (no limitation on type or extent)	No educational intervention
Comparison	There will be no restriction on the comparator used in eligible studies (not applicable for the research objective, if applicable: Intervention methods described as comparators in the included studies)	
Outcome	Targeting one or multiple learning outcomes related to competencies for advancing sustainability: Key competencies in sustainability: Planning competencies: systems‐thinking; futures‐thinking; values‐thinking, strategies‐thinking Key professional competencies: inter‐ and intra‐personal competencies Implementation competence Integration competence Disciplinary competencies General competencies (e.g., critical‐thinking, creativity, learning and self‐efficacy) Professional Competencies (e.g., communication skills and project management)	Learning objectives and learning outcomes do not have a focus on content related to sustainability
Study design	Any empirical research, including experimental, quasi‐experimental and observational studies involving education interventions targeted at dietitians/nutrition professionals. Programs or initiatives targeted at dietitians/nutrition professionals. Qualitative, quantitative and mixed‐method studies.	
Additional criteria	Published in German or English	Published in languages other than English or German

On 18 June 2024, relevant literature was searched in PubMed incl. MEDLINE, SCOPUS and Web of Science (Core Collection: Conference Proceedings Citation Index, Science Citation Index Expanded, Social Sciences Citation Index and Emerging Sources Citation Index) and ProQuest. Additionally, a free search in Google Scholar was conducted to identify unindexed or non‐peer‐reviewed studies, minimising potential selection or publication bias. Reference lists of included studies were hand‐searched for further relevant studies. The search strategy combined text words and keywords from four categories (1) dietitians and nutrition professionals, (2) educational intervention, (3) sustainability and (4) competence, with truncation applied for word stems to ensure comprehensive coverage. Search terms were adapted per database and are detailed in Appendix 1, Supporting Information S1 (Tables A1–A4).

**FIGURE 1:**
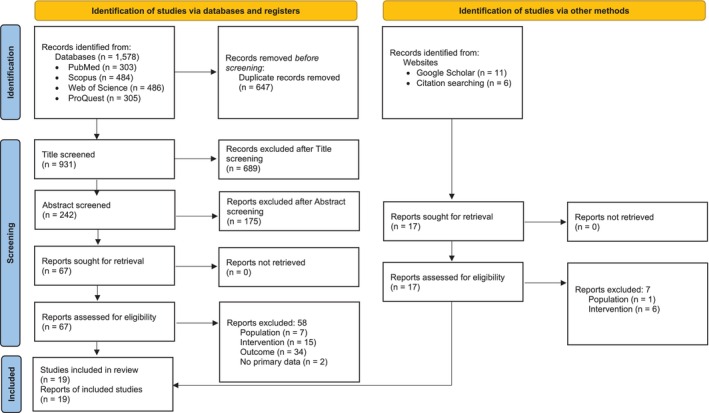
Flow diagram of literature search and study selection according to Preferred Reporting Items for Systematic Reviews and Meta‐Analysis 2020 guidelines.

Citavi 6 was used for reference management during the screening process. The three‐staged screening of titles, abstracts and full texts was performed by the first author, with eligibility assessed using the predefined inclusion criteria. Irrelevant and duplicate publications were removed. Any records which were unclear were discussed with the second author. The selection process is depicted in a Preferred Reporting Items for Systematic Reviews and Meta‐Analyses flow chart, as shown in Figure 1. Data collection was carried out by the first author using the software MAXQDA 2022 and Microsoft Excel (2018 version). The data items collected were: study characteristics (author, title, year and country of origin), population (profession and number of participants), characteristics of the intervention (title, type, structure, duration, teaching methods, pedagogical concept, lecturers, content, learning objectives, learning outcomes and evaluation method) and summary of the findings.

The methodological quality of included studies was assessed using critical appraisal tools from the Johanna Briggs Institute for case report,^23^ qualitative research^24^ and quasi‐experimental studies.^25^ Quantitative descriptive and mixed method studies were evaluated using the Mixed Methods Appraisal Tool.^26^ The first author conducted the quality assessment. Any uncertainties were discussed with the second author.

Results were synthesised narratively, with data systematically extracted in text form and tabulated. Studies were categorised based on the defined data items, to allow a structured comparison of findings. Within the studies, reporting of the educational intervention varied, with incomplete information on pedagogical concepts, lecturers, learning objectives, outcomes and evaluation methods due to the particular research objective and study design. Studies were included if sufficient data allowed mapping to Redman and Wiek's^18^ and Lozano et al.'s^21^ frameworks. Sustainability competencies were mapped based on the description of learning outcomes, learning objectives, structure and content of the intervention. Competencies were mapped if explicitly stated in this description according to Redman and Wiek's^18^ definition; otherwise, learning outcomes or course content were analysed to determine if they revealed a meaning that described a competence. Pedagogical approaches, as defined by Lozano et al.,^21^ were identified based on teaching methods, structure and lecturers. The information on the content of the intervention was relevant for matching the approaches eco‐justice and community as well as supply‐chain/life cycle analysis and traditional ecological knowledge.

Due to the heterogeneity in study designs (including qualitative studies and case reports) and varying outcome measures in quasi‐experimental intervention studies, quality assessment using the Grading of Recommendations Assessment, Development and Evaluation methodology, as outlined in the protocol, was not feasible.

## 3 RESULTS

The database search identified 1578 publications, of which 647 were duplicates that were removed. After screening the titles and abstracts, the full text of 67 publications was screened. Free search in Google Scholar and citation searching identified an additional 17 publications, of which the full text was screened.

Thirty‐four studies were excluded since they focused on a non‐sustainability‐related outcome parameter. A further 21 studies were excluded as they do not focus on an educational intervention, but only examined the level of knowledge or skills, students' perceptions of sustainability, recommendations for future teaching or the inclusion of sustainability into the curriculum; they did not include information on how this is done. Eight records did not focus on dietitians or other nutrition professionals. Two studies were excluded because they were not primary research. Nineteen studies met the inclusion criteria and were considered for this study.^27^, ^28^, ^29^, ^30^, ^31^, ^32^, ^33^, ^34^, ^35^, ^36^, ^37^, ^38^, ^39^, ^40^, ^41^, ^42^, ^43^, ^44^, ^45^ The literature search and selection process are shown in Figure 1.

The methodological quality of the included studies was heterogeneous. Case reports met between three and six of eight criteria, qualitative studies between 6 and 9 of 10, and quasi‐experimental studies between three and seven of nine, according to the respective Johanna Briggs Institute checklists. Mixed‐methods studies fulfilled six of seven criteria, and the quantitative study five of seven, based on the Mixed Methods Appraisal Tool. Detailed appraisal results are provided in Appendix 2, Supporting Information S1 (Table A5).

As detailed in Table 2, the identified studies were conducted in the United States (*n* = 9), Canada (*n* = 5), Spain (*n* = 2), Australia (*n* = 2) and New Zealand (*n* = 1). Study designs included qualitative studies (*n* = 6), case study reports (*n* = 5), quasi‐experimental studies (*n* = 5), mixed‐methods studies (*n* = 2) and a quantitative cross‐sectional study (*n* = 1). The study populations primarily comprised undergraduate and graduate students enrolled in nutrition, dietetics and other food‐related disciplines, such as agricultural sciences or food science and technology. Additionally, four interventions were also open to students from other fields, including, for example, applied economics, health sciences or public administration. The interventions varied widely in format and duration. Six studies refer to one or more university courses; 13 studies report on one or more individual learning activities that were incorporated into an existing course or offered as extracurricular components.

**TABLE 2:** Characteristics of interventions for dietetic and nutrition students: description of reviewed studies.

Study and country	Study design	Population	Type and duration	Teaching methods and activities	Content
Brekken et al.^28^ United States (Oregon State University, University of Minnesota, University of Vermont)	Case study report (description of the intervention)	First course: *n* = 30–40, undergraduate and graduate students (Agricultural Sciences, Food in Culture & Social Justice) Second course: *n* = 55 students (Food Systems undergraduate major, Applied Economics, Agricultural & Food Business Management) Third course: *n* = 5–10 graduate students (Food Systems, Applied Economics, Natural Resources, Plant & Soil Science, Public Administration)	University‐level courses: first: elective or required (varies by program), second: required Third: elective *Duration*: unknown	Deductive case studies: food system indicators, retail analysis project, market failure and policy concept application experiential learning: guest speaker talks, virtual field trips, food diary, student‐led workshop reflective narrative learning: reflection papers, food stories, in‐class reflection, discussion system dynamics simulations and scenarios: casual loop diagram, food system diagram, modelling population dynamics, modelling herbicide resistance, food system history inductive case studies: policy brief, science, technology and society paper, food system problem term project, institutional food service case study	Food systems and systems‐thinking with an economic lens
Bustamante et al.^27^ Spain (University of Basque Country)	Pilot study (description of the intervention)	*n* = 17; Students (Human Nutrition and Dietetics)	Last year subject *Duration*: unknown	Lectures, practical cooking activity including group work and written reports, seminars including critical reading activity, group discussion and practical cases of menu planning	Sustainability and collective restauration
Carino et al.^29^ Australia	Cross‐sectional study (qualitative content analysis of documented online course outlines and online survey)	Third‐year undergraduate nutrition students *n* = unknown	Compulsory module *Duration*: 1 semester	Scenario‐based‐learning activity including discussions and reflection, Global Diary Guidelines Comparison incl. reflection, eco‐friendly food challenge incl. reflection report and developing of a resource to motivate fellow students, Food System Audit incl. written report and governmental brief	Food supply system; sustainable food system
Fox^30^ Canada (St. Francis Xavier University)	Case study report (description of the intervention)	*n* = 15; students (Bachelor of Science in Human Nutrition)	Optional project that is incorporated into a fourth‐year undergraduate nutrition course *Duration*: 4 months	Project‐based learning activity (group work): creating project plan based on a conference recommendation, student presentations, written report	Food security and climate change
Hege et al.^31^ United States (Iowa State University)	Cross‐sectional study (online surveys)	*n* = 140, distance dietetic interns	Six out of 13 learning activities of the sustainable food system curriculum incorporated into internship *Duration*: 40–65 h	Literature research and seminar presentation interview and write a newsletter article Personal reflection and student presentation food waste audit in an institutional setting healthier food retail assessment Investigation how food policy is addressed in your community and action plan development (group work)	Sustainable food systems, school foodservice, food waste, food retail, food policy, nutrition care process
Holik et al.^32^ United States (mid‐sized regional comprehensive university)	action‐based research using mixed methods for post‐intervention data collection	*n* = 196; nutrition and dietetic students	Two experiential learning activities within existing course *Duration*: min. 4 h	Experiential learning activity (shadowing an registered dietitian), active learning exercises	Food service management
Innes et al.^33^ New Zealand (University of Otago)	Intervention study (pre‐/post design)	*n* = 55; students of Human Nutrition	Two‐week‐module, part of an undergraduate second‐year course *Duration*: 2 weeks	Lectures, documentaries, problem‐solving group activities, self‐directed learning outside the class	Environmental impact of food production and consumption
Knobloch et al.^34^ United States (Texas A&M University, Purdue University, Ohio State University)	One‐group pre‐experimental case study (pre‐/post design)	*n* = 32 (11 doctoral students, 20 master's students, 1 undergraduate student); (Agriculture & Natural Resources, Business & Economics, Health, Education & Communication, Food Science & Technology, Human Nutrition)	Interdisciplinary graduate course *Duration*: unknown	Online modules (content presented as case study, specific problem or issue) synchronous and asynchronous discussions, phenomenon‐based learning (project work in interdisciplinary groups): (a) community food security assessment (incl. own data collection and research for community profile, place‐based learning activity, interviews), (b) expert interview, video production (multimedia storytelling), (c) create an e‐learning module	Food and nutritional security, hunger and sustainability
Maher and Burkhart^35^ Australia (University of the Sunshine Coast)	Intervention study (qualitative approach)	*n* = 143; Bachelor of Nutrition, Nutrition & Dietetics (majority) and other programs	Eco‐friendly food challenges (four options, students choose one); part of a compulsory course; required course for: Nutrition and Nutrition & Dietetics students; elective for others *Duration*: 3 weeks	Lecture, tutorials for instructions, eco‐friendly food challenge, blogging (reflection), group feedback session	Sustainable food systems and dietary practices
Matthews^36^ Canada (Brescia University College in Western University)	Case study report (description of the intervention)	*n* = 165 undergraduate students (Food and Nutritional Sciences)	Project within first‐year course with 25% of the lectures about sustainability *Duration*: unknown	Lectures, project work (food system assessment) incl. own data collection and analysis, site visits, groupwork; photovoice activity and group discussion	Human nutrition, food security, world hunger, agriculture, sustainability, food system assessment
Meyer et al.^37^ United States (University of Colorado Colorado Springs)	Intrinsic case study (mixed methods)	*n* = 75 (43 undergraduate, 15 graduate students, 15 faculty, 2 staff); nutrition majors	Student‐run enterprise with a student‐developed sourcing approach as a co‐curricular activity embedded in optional practicum rotation *Duration*: unknown (continuous project)	Hands‐on learning activity (managing and work in a food establishment incl. menu design, procurement, work on farm, cooking, serving with food literacy and taste education, recovering/reusing/composting), peer‐teaching	Local and sustainable food production and consumption
Miller et al.^38^ United States (Two universities in Ohio)	Qualitative study	*n* = 26; undergraduate dietetic students, enrolled in community nutrition courses	Didactic teaching combined with an experiential learning activity and reflections (required) *Duration*: farm tour 5 h total duration: unknown	Online modules as videos (didactic teaching); experiential‐learning activity (=service‐learning activity: farm tour, discussion, hands‐on work [planting, weeding, filling beds and harvesting produce]); written reflection; group discussion	Sustainable food systems via university‐based farm tour
Navarro et al.^39^ Spain (University of the Basque Country)	Quasi‐experimental intervention study (pre‐/post‐design)	*n* = 49; students of Human Nutrition and Dietetics	Ten learning activities integrated in seven courses during the 4‐year human nutrition and dietetics degree *Duration*: unknown	Active and collaborative learning strategies: Case method, group activities, role playing/double convergence divergence	Sustainability within the topic of each course
Pabani et al.^40^ Canada (Mount Saint Vincent University)	Exploratory study (qualitative approach)	*n* = 5; post‐secondary dietetic students	Research project within dietetic placement *Duration*: from 3 month to 4 or more years depending on nature of placement	Project‐based learning: research projects guided by concept of community‐based participatory research (plan and facilitate research trainings, analyse and disseminate research findings, develop tools to support research partners)	Household food insecurity and community food security
Pontikis et al.^41^ United States (California State University, Northridge)	Case study report (description of the intervention)	*n* = unknown; Family and Consumer Science, Nutrition, Dietetics and Food Science students	Four courses across a Nutrition and Dietetics program *Duration*: unknown	Debate; hands‐on projects with community partners and organisations; lectures; weekly written assignments, final project (developing a community service‐learning initiative)	Environmental impact of diets and food production (courses 1–3) sustainability within Family and consumer sciences (course 4)
Ruhl and Lordly^42^ Canada (Mount Saint Vincent University)	Descriptive study (qualitative approach)	*n* = 41 students, *n* = 2 lab instructors, *n* = 3 student volunteers; students of a nutrition program	Community garden laboratory (1 of 11 labs) as part of an introductory foods course within nutrition program; *Duration*: unknown	Lecture, experiential learning activity: visit community garden incl. reading activity, reflection and discussion within small groups and in‐kitchen cooking activity incl. discussion	Community garden, food sustainability, growing and harvesting food
Shafto et al.^43^ United States (University of Minnesota)	Intervention study (pre‐/post‐design)	*n* = 65 health science graduate students, among others Nutrition/Dietetic Students (*n* = 4 [6,2%])	1‐credit graduate level course, required for the Integrative Health and Healing Doctorate of Nursing Practice students, for others elective *Duration*: 18 h	Food Story activity: students share own experiences culinary experiences (practice cooking skills) food skills practice (reading nutrition labels, identifying macronutrients in food) culinary assignments dietary patterns education through recipe illustration of concepts discussed in didactic segments students assignments (personal reflection, mindful eating experiences, review of literature and online discussion forum). Food‐mood journal interdisciplinary group discussion case project: group work (with peer‐teaching opportunity), creating a video, submission of an academic paper	Clinically oriented nutrition concepts with regard to health and the food system; culinary skills
Spiker et al.^44^ United States (Iowa State University, Oregon Health & Science University, Northern Illinois University, University of Kentucky)	Pilot intervention study (pre‐/post‐design)	First intervention: *n* = 38 pre‐test; *n* = 10 post‐test second intervention: *n* = 28 pre‐test; *n* = 5 post‐test dietetics interns and graduate students	Two interactive webinar series as optional learning activities beyond required curricula *Duration*: 3 months	Case studies, practice activities (impact analysis, brainstorming of strategies to change food system), jigsaw technique (with group discussions)	Systems‐thinking and connection between dietetic practice and sustainable food systems
Wadsworth et al.^45^ Canada (St. Francis Xavier University)	Case study (qualitative approach)	*n* = 47; students of human nutrition (two courses: Community Nutrition (*n* = 30) and Food Availability (*n* = 17))	Experiential learning activity: 18 service‐learning projects in two upper‐level courses over the 3 years of the study program *Duration*: unknown	Service‐learning activities (project work with community partners), reflection (written and verbal, final report)	Local food systems

Interventions were mapped to sustainability competencies defined by Redman and Wiek^18^ based on content, learning objectives and outcomes (Table 3). With regard to the central key competencies of the framework, systems‐thinking (*n* = 16), strategies‐thinking (*n* = 15), implementation competence (*n* = 12) and values‐thinking (*n* = 11) were addressed most frequently. Fewer studies addressed inter‐personal competence (*n* = 8), integration competence (*n* = 6) and futures‐thinking (*n* = 4). Intra‐personal competence was not covered in any intervention. Complementary competencies (disciplinary, general and professional competence) were addressed in all interventions.

**TABLE 3:** Overview of the addressed sustainability competencies based on the definition by Redman and Wiek.^18^

	Systems‐thinking	Futures‐thinking	Values‐thinking	Strategies‐thinking	Inter‐personal competence	Implementation competence	Integration competence	Intra‐personal competence	Disciplinary competencies	General competencies	Professional competencies
Brekken et al.^28^	x	x	x	x	x				x	x	x
Bustamante et al.^27^			x	x		x	x		x	x	x
Carino et al.^29^	x	x	x	x		x			x	x	x
Fox^30^	x		x	x					x	x	x
Hege et al.^31^	x		x	x	x	x	x		x	x	x
Holik et al.^32^	x		x			x			x	x	x
Innes et al.^33^				x					x	x	x
Knobloch et al.^34^	x	x	x		x				x	x	x
Maher and Burkhart^35^	x		x	x		x	x		x	x	x
Matthews^36^	x			x					x	x	x
Meyer et al.^37^	x		x	x	x	x	x		x	x	x
Miller et al.^38^	x					x			x	x	x
Navarro et al.^39^	x	x	x	x					x	x	x
Pabani et al.^40^	x			x	x	x	x		x	x	x
Pontikis et al.^41^	x			x					x	x	x
Ruhl and Lordly^42^	x			x	x	x			x	x	x
Shafto et al.^43^				x	x	x	x		x	x	x
Spiker et al.^44^	x					x			x	x	x
Wadsworth et al.^45^	x		x	x	x	x			x	x	x

The mapping of data to pedagogical approaches from Lozano et al.^21^ is shown in Table 4. Most commonly used approaches were project and/or problem‐based learning (*n* = 17), lecturing (*n* = 11), place‐based environmental education (*n* = 9), Case studies and supply‐chain/life cycle analysis (*n* = 7 each). According to Lozano et al.^21^ project and/or problem‐based learning and place‐based environmental education are highly likely to foster sustainability competencies, enhancing interdisciplinary work, anticipatory thinking, critical thinking and analysis, inter‐personal relations and collaboration (project and/or problem‐based learning) as well as personal involvement (place‐based environmental education). Whereas lecturing, case studies and supply‐chain/life cycle analysis, also most frequently used, are the least likely to develop competencies.^21^

**TABLE 4:** Summary of methods applied, as defined by Lozano et al.^21^

References	SC	ITT	L	MACM	PPBL	CSL	JIT	PAR	EJC	PBEE	SC/LCA	TEK	Additional methods
Brekken et al.^28^	x		x	x	x					x	x	x	Reflective narrative learning activities (reflection paper, share a food story, in‐class reflections, intensive discussion)
Bustamante et al.^27^	x		x		x								Group discussion
Carino et al.^29^	x				x					x	x		Discussion and reflection included in PPBL activities
Fox^30^					x							x	–
Hege et al.^31^					x					x			Personal reflection
Holik et al.^32^										x	x		–
Innes et al.^33^			x		x								–
Knobloch et al.^34^	x	x	x		x				x	x			Synchronous and asynchronous discussions
Maher and Burkhart^35^			x		x								Reflection via blogging included in the PPBL activity
Matthew^36^			x		x					x	x		PPBL included group discussion for reflection
Meyer et al.^37^					x	x				x	x		Peer‐teaching
Miller et al.^38^			x			x				x			Written reflections using given questions and prompts; group discussion
Navarro et al.^39^	x				x						x		–
Pabani et al.^40^					x	x		x	x				–
Pontikis et al.^41^		x	x		x	x							Debate
Ruhl and Lordly^42^			x		x	x			x	x	x		Reflection, discussion within small groups
Shafto et al.^43^	x	x	x		x								Personal reflection, online discussion forum, group discussion group work with peer‐teaching
Spiker et al.^44^	x	x	x		x		x						Group discussion
Wadsworth et al.^45^					x	x				x			Written and verbal reflection

Abbreviations: CSL, community service learning; EJC, eco‐justice and community; ITT, interdisciplinary team teaching; JIT, Jigsaw/Interlinked Teams; L, lecturing; MACM, mind and concept maps; PAR, Participatory Action Research; PBEE, place‐based environmental education; PPBL, project and/or problem‐based learning; SC/LCA, supply‐chain/life cycle analysis; TEK, traditional ecological knowledge.

Fewer interventions used interdisciplinary team teaching (*n* = 4), community service learning (*n* = 6) and eco‐justice and community (*n* = 3), all of which are highly likely to develop sustainability competencies. According to Lozano et al.,^21^ Community service learning fosters anticipatory thinking, empathy and change of perspective, strategic action and personal involvement. Interdisciplinary team teaching promotes interdisciplinary work, and eco‐justice and community encourages interdisciplinary work, justice, responsibility and ethics, empathy and change of perspective, personal involvement and tolerance for ambiguity and uncertainty.^21^

The least frequently used approaches were Jigsaw/Interlinked Teams (*n* = 1), Mind and concept maps (*n* = 1), Participatory Action Research (*n* = 1), traditional ecological knowledge (*n* = 2). Jigsaw/Interlinked Teams and Mind and concept maps are both likely to develop sustainability competencies, especially empathy and change of perspective. In Lozano, R. et al.'s^21^ framework, traditional ecological knowledge and Participatory Action Research are categorised as ‘maybe’ in terms of their potential to develop sustainability competencies, indicating neither high nor low likelihood.^21^

Studies included additional teaching strategies beyond Lozano et al.'s^21^ framework, in particular various forms of reflective learning (*n* = 9), discussion and debate (*n* = 9), and peer‐teaching, which was not Jigsaw/Interlinked Teams (*n* = 2), as detailed in Table 4.

## 4 DISCUSSION

This systematic review, based on 19 studies, examines how educational interventions for dietitians and nutrition professionals foster sustainability competencies, following the definition by Redman and Wiek.^18^ Additionally, it explores the pedagogical approaches employed and how these approaches facilitate the development of sustainability competencies, following the framework proposed by Lozano, R. et al.^21^ Although several studies have examined the integration of sustainability into the education of dietitians and nutrition professionals and assessed its inclusion at different cognitive levels,^13^, ^14^, ^16^, ^17^, ^29^, ^46^ no studies could be found that have analysed the extent to which sustainability competencies are addressed. This gap limits our understanding of whether future dietitians and nutrition professionals are sufficiently equipped to integrate sustainability principles into their professional practice.

Analysis of sustainability competencies, based on Redman and Wiek's^18^ definition, revealed that systems‐thinking, strategies‐thinking, values‐thinking and implementation competence were the most frequently addressed key competencies. General, professional, and disciplinary competencies were considered in all interventions. However, less than half of the interventions explicitly addressed futures‐thinking, intra‐personal, inter‐personal and integration competencies. Insufficient coverage of all sustainability competencies within individual interventions can lead to gaps in students' overall competence development. To ensure holistic sustainability education, teaching approaches should aim to address a broad range of competencies rather than emphasising isolated aspects.^19^

Futures‐thinking competence is crucial for anticipating a sustainable future of our food system and the impact of sustainability measures.^18^ Its limited coverage in only four studies is concerning, as understanding a desirable future is fundamental to transforming the food system. This aligns with findings from a scoping review showing low integration of anticipatory competence in higher education^47^ and an analysis highlighting a lack of futures‐thinking in primary school education.^48^ Approaches such as scenarios, simulation or visioning methods can be used for teaching futures‐thinking.^49^ For example, in the study by Navarro et al.,^39^ students were required to predict near‐future scenarios based on whether a proposed intervention was implemented or not, and to anticipate the potential consequences of a designed intervention plan.^39^

Intra‐personal competence, which includes self‐care, stress management and coping with negative experiences, has not been integrated into any intervention. However, this is particularly relevant for dietitians and nutrition professionals, who, as a caring and helping profession, face high stress and burnout risks.^50^ Moreover, the issue of climate change can generally trigger negative emotions that are associated with insomnia symptoms and mental health concerns, for example,^51^ underscoring the importance of addressing intra‐personal competence. For example, mindfulness techniques (e.g., awareness walk, meditation or yoga combined with reflective discussions) have been shown to be effective self‐care strategies for counsellors‐in‐training.^52^ Inter‐personal competence, addressed in only eight studies, involves collaborating in a team, bringing together everyone's knowledge and skills to tackle complex tasks,^53^ such as creating a sustainable food system. Given the diverse settings dietitians and nutrition professionals work in, including interactions with different stakeholders such as physicians or nurses, interdisciplinary and transdisciplinary collaboration is essential for contributing to a sustainable food system.^6^, ^8^ As part of the inter‐personal competence, it is important to learn how to engage different stakeholders,^53^ as the transformation of our food system requires the collective participation of all relevant agents.^5^ This highlights the need to incorporate inter‐personal competence in dietetic education, which would be possible by using approaches for effective communication, listening or approaches for trust building, including non‐judgmental interactions.^49^ For example, collaborative learning enhances students' group collaboration skills. Central to the success of collaborative learning is targeted teacher guidance, such as providing feedback on students' collaboration progress and assisting students in resolving conflicts.^54^

Lastly, integration competence, which implies a combination of two or more of the key competencies, collective problem‐solving to develop strategies and implement them^18^, ^55^ was addressed in only six studies. Although this more complex competence may be challenging to teach, it is essential for fostering sustainability competencies holistically. Recommended methods for teaching integration competence include transition management/governance, organisational change management or transformational planning methodology.^49^

The analysis of pedagogical approaches used in the reviewed studies, based on Lozano, R. et al.,^21^ reveals that project and/or problem‐based learning, lecturing and place‐based environmental education were the most frequently employed. The frequent use of practical approaches like place‐based environmental education and project and/or problem‐based learning is a favourable finding, given their strong potential to foster sustainability competencies.^21^ Project and/or problem‐based learning in sustainability education enhances student‐centred learning by engaging students with complex, real‐world challenges, emphasising solution‐oriented thinking, teamwork and stakeholder collaboration.^56^, ^57^ Real‐world learning opportunities like Place‐based environmental ducation bridge knowledge with practical action, fostering sustainability competencies through critical reflection, problem‐solving, collaboration and accountability in real‐world contexts.^58^ Lecturing, supply‐chain/life cycle analysis and case studies, while frequently used in the interventions, are least effective in fostering sustainability competencies.^21^ However, this does not imply that these methods should be discarded; rather, they should be adapted or integrated with complementary pedagogical approaches to enhance competency development.^21^ Analysis of the identified interventions indicates that these methods were consistently combined with at least one practical, community‐oriented approach, including project and/or problem‐based learning, place‐based environmental education or community service learning, ensuring a more comprehensive learning experience. Even if some approaches hold potential for promoting sustainability competencies, combining them is a good way to effectively address all competencies.^21^ Practice‐oriented and interactive teaching methods, as opposed to traditional lecturing, enhance student engagement and sustainability awareness. However, educators face challenges in adopting these pedagogical approaches, particularly due to necessary preparation.^59^

Although effective, other practical and community‐based approaches such as community service learning and eco‐justice and community were underutilised, despite their high potential for developing sustainability competencies.^21^ These approaches should be more widely implemented, as sustainability education is most effective when learners are actively involved in designing and implementing school or community projects that address climate‐related challenges.^60^

The least frequently used approaches were mind and concept maps, Jigsaw/Interlinked Teams, Participatory Action Research and traditional ecological knowledge. A possible explanation for low utilisation in teaching could be challenging practical application. While mind maps are relatively simple tools for structuring knowledge, concept maps prove to be more demanding, as their use requires intensive familiarisation and training, making classroom integration more challenging.^61^ The limited use of Jigsaw/Interlinked Teams may stem from challenges like varying student engagement levels, differing learning speeds and the need for time‐intensive and complex planning.^62^ Nevertheless, according to Lozano et al.,^21^ Mind and concept maps and Jigsaw/Interlinked Teams are highly likely to develop empathy and change of perspective. This competence may be linked to a subset of intra‐personal competence, as defined by Redman and Wiek,^18^ which includes reflection, empathy and respect for identity, commitment and feelings. As intra‐personal competence was not explicitly addressed in any intervention, greater use of these approaches is warranted. Furthermore, integrating traditional ecological knowledge into education poses additional challenges of authentically representing Indigenous voices without instrumentalisation. Educators must navigate the risks of cultural appropriation, oversimplification of complex knowledge systems and the tokenisation of Indigenous perspectives, making this a particularly demanding task.^63^

Overall, effectively addressing sustainability competencies in teaching and using appropriate methods depends on whether educators are adequately trained and have sufficient resources. These resources include time, institutional support and motivation^19^—factors that, while not the focus of this review, are nonetheless critical for successful implementation. A lack of structural support—for example, teaching materials, time, institutional frameworks—can hinder the integration of sustainability into teaching.^59^ It is notable that a 2011 survey already found that dietetic educators are generally interested in incorporating sustainability into their teaching, but are concerned about how to do so. Providing concrete resources, such as curriculum guidelines, assignment templates, project ideas or reading lists may reduce perceived barriers.^64^ Additionally, the same study reported educators' concerns about students' interest in learning about sustainability.^64^ Limited intrinsic student motivation may pose an additional barrier to successful integration of sustainability into educational practice.^59^ However, initial research findings suggest that nutrition students recognise the importance of sustainability^65^ and are generally motivated to act sustainable.^11^

The results of this study may be limited by several aspects. First, the reviewed studies were conducted across diverse cultural, curricular and institutional contexts, which may limit the generalisability of the findings. Second, this review's screening and data extraction were conducted by one author, which may increase the risk of selection and information bias. To mitigate this risk, ambiguous cases were discussed with the second author to reach a consensus. Third, a potential language and reporting bias may exist, as only articles published in German or English were included. Additionally, selective reporting within studies, evidenced by varying levels of detail in the reporting of interventions across included studies, could not be fully assessed, potentially introducing a reporting bias that may affect the interpretation of the results. Fourth, although differences in the study quality of included studies were noted, this was not a primary concern, as the aim was to analyse and describe existing interventions rather than assess their effectiveness. Consequently, this study cannot draw conclusions about the actual effect of the interventions or whether their duration was sufficient to foster sustainability competencies. This highlights a need for further research to evaluate the impact of educational interventions on sustainability competency development and to establish a validated measurement tool based on Redman and Wiek's^18^ framework. Furthermore, as the review focused on scientific publications, it remains unclear to what extent sustainability competencies are embedded in professional training standards, which falls outside the scope of these methods. While this review indicates encouraging progress in dietitians and nutrition professionals' sustainability education, it is essential that sustainability competencies are integrated holistically into all curricula.^19^ While dietetic educators are guided by national competency standards, this analysis remains relevant by providing guidance for advancement of their teaching activities. It identifies opportunities for educators on how to complement and further develop current teaching practices to support sustainability education. Moreover, the findings may serve as a foundation for future revisions of professional standards by highlighting areas for improvement.

A further limitation is that the underlying frameworks by Redman and Wiek^18^ and Lozano et al.^21^ are not directly comparable due to differing definitions of sustainability competencies. Thus, no definite conclusion can be drawn about how pedagogical approaches classified by Lozano et al.^21^ contribute to sustainability competencies defined by Redman and Wiek.^18^ This discrepancy emphasises the need for standardised definitions of sustainability competencies across frameworks to enable more meaningful analysis of the relationship between teaching methods and sustainability competencies. Aligning pedagogical approaches with defined sustainability competencies remains crucial, for which the framework by Lozano et al.^21^ is currently a useful tool. Lastly, although there is some overlap with studies included in McCormack et al.^16^ due to similar search terms, this review differs by providing a more in‐depth focus on nutrition education. McCormack et al.^16^ conducted a scoping review investigating teaching and evaluation strategies, as well as learning outcomes, applying the Kirkpatrick‐Barr framework to guide analysis.

Concluding our results indicate how sustainability competencies are currently addressed in dietitians and nutrition professionals' education. While many competencies, as defined by Redman and Wiek,^18^ are already integrated, inter‐personal, integration, intra‐personal and futures‐thinking competencies in particular remain underrepresented. To prepare dietitians and nutrition professionals to support the transition towards a sustainable food system, sustainability competencies should not only be developed individually within separate learning activities. Instead, a holistic and coordinated integration across training programs is essential, as all competencies are essential for driving sustainability transformations.^18^ In case of limited time and capacity, specialisation may be appropriate. Students may develop deep expertise in one or two sustainability competencies while building a solid foundation in the others. Expectations should be aligned with the academic level, with varying degrees of depth at undergraduate, master's or doctoral programs.^66^ The frequent use of practical approaches such as place‐based environmental education and project and/or problem‐based learning is encouraging, given their strong potential to promote sustainability competencies, as outlined by Lozano et al.^21^ Notably, less effective methods like traditional lectures were combined with such practical approaches, enhancing overall impact. Such combinations offer a valuable strategy for revising existing training concepts, by examining how currently used methods can be beneficially supplemented with more effective approaches to better support sustainability learning. Overall, embedding sustainability competencies and suitable pedagogical approaches into dietitians and nutrition professionals' education will require time and systemic effort. Consolidating standards across courses, programs and institutions is a complex process that requires sustained coordination and collaboration among faculty, curriculum planners and institutional leaders.^66^

## AUTHOR CONTRIBUTIONS

RL was responsible for the conceptualisation of the study, literature search, screening, data synthesis and analysis and drafting the original manuscript. TB contributed to the study design and methodology, provided supervision throughout the entire research process and critically reviewed and edited the manuscript. Both authors approved the final version of the manuscript.

## FUNDING INFORMATION

The authors declare that no financial support was received for the conduct of this work or the preparation of the manuscript. The publication is partly funded by the Open Access Publishing Fund of the University of Bayreuth.

## CONFLICT OF INTEREST STATEMENT

The authors declare no conflicts of interest related to this work. No relevant financial or non‐financial relationships, activities or potential conflicts of interest exits that could have influenced the research, analysis or conclusions presented in this manuscript.

## Supporting information


**Data S1.** Supporting Information.

## Data Availability

The data that support the findings of this study are available from the corresponding author upon reasonable request.
