# Supplementary material for: Enhancing sustainability competencies: A systematic review of educational interventions for dietitians and nutrition professionals
Source: Nutr Diet. 2025 Sep 17;82(5):501–17. doi: 10.1111/1747-0080.70046 (PMC12583897; doi:10.1111/1747-0080.70046)
Supplement: Supplementary file 1 — Data S1. Supporting Information. [file NDI-82-501-s001.docx]

# Appendix

## Appendix 1 Search strategy

**PudMed**

Table A1: search in PubMed

| # | Search Query | Results | Date Run |
| --- | --- | --- | --- |
| #1 Population | "nutritionist*"[Title/Abstract] OR "dietician*"[Title/Abstract] OR "dietitian*"[Title/Abstract] OR "nutrition* professional*"[Title/Abstract] OR "dietetic* student*"[Title/Abstract] OR "nutritionists"[MeSH Terms] OR "nutrition* student*"[Title/Abstract] | [14,988](https://pubmed.ncbi.nlm.nih.gov/?term=%22nutritionist%2A%22%5BTitle%2FAbstract%5D+OR+%22dietician%2A%22%5BTitle%2FAbstract%5D+OR+%22dietitian%2A%22%5BTitle%2FAbstract%5D+OR+%22nutrition%2A+professional%2A%22%5BTitle%2FAbstract%5D+OR+%22dietetic%2A+student%2A%22%5BTitle%2FAbstract%5D+OR+%22nutritionists%22%5BMeSH+Terms%5D+OR+%22nutrition%2A+student%2A%22%5BTitle%2FAbstract%5D&sort=relevance&ac=no) | 18.06.2024 |
| #2 Intervention – formal | "educat*"[Title/Abstract] OR "train*"[Title/Abstract] OR "curricul*"[Title/Abstract] OR "learn*"[Title/Abstract] OR "program*"[Title/Abstract] OR "teach*"[Title/Abstract] OR "pedagog*"[Title/Abstract] OR "course"[Title/Abstract] OR "educational status"[MeSH Terms] OR "education"[MeSH Terms] | [3,875,244](https://pubmed.ncbi.nlm.nih.gov/?term=%22educat%2A%22%5BTitle%2FAbstract%5D+OR+%22train%2A%22%5BTitle%2FAbstract%5D+OR+%22curricul%2A%22%5BTitle%2FAbstract%5D+OR+%22learn%2A%22%5BTitle%2FAbstract%5D+OR+%22program%2A%22%5BTitle%2FAbstract%5D+OR+%22teach%2A%22%5BTitle%2FAbstract%5D+OR+%22pedagog%2A%22%5BTitle%2FAbstract%5D+OR+%22course%22%5BTitle%2FAbstract%5D+OR+%22educational+status%22%5BMeSH+Terms%5D+OR+%22education%22%5BMeSH+Terms%5D&sort=relevance&ac=no) | 18.06.2024 |
| #3 Intervention – Content | "sustainab*"[Title/Abstract] OR "environment*"[Title/Abstract] OR "climate change"[Title/Abstract] OR "planetary health"[Title/Abstract] OR "climate change"[MeSH Terms] OR "environment"[MeSH Terms] | [2,846,937](https://pubmed.ncbi.nlm.nih.gov/?term=%22sustainab%2A%22%5BTitle%2FAbstract%5D+OR+%22environment%2A%22%5BTitle%2FAbstract%5D+OR+%22climate+change%22%5BTitle%2FAbstract%5D+OR+%22planetary+health%22%5BTitle%2FAbstract%5D+OR+%22climate+change%22%5BMeSH+Terms%5D+OR+%22environment%22%5BMeSH+Terms%5D&sort=relevance&ac=no) | 18.06.2024 |
| #4 Outcome | "competence*"[Title/Abstract] OR "literacy"[Title/Abstract] OR "capab*"[Title/Abstract] OR "knowledge"[Title/Abstract] OR "understand*"[Title/Abstract] OR "skill*"[Title/Abstract] OR "think*"[Title/Abstract] | [3,311,852](https://pubmed.ncbi.nlm.nih.gov/?term=%22competence%2A%22%5BTitle%2FAbstract%5D+OR+%22literacy%22%5BTitle%2FAbstract%5D+OR+%22capab%2A%22%5BTitle%2FAbstract%5D+OR+%22knowledge%22%5BTitle%2FAbstract%5D+OR+%22understand%2A%22%5BTitle%2FAbstract%5D+OR+%22skill%2A%22%5BTitle%2FAbstract%5D+OR+%22think%2A%22%5BTitle%2FAbstract%5D&sort=relevance&ac=no) | 18.06.2024 |
| #5 | #1 AND #2 AND #3 AND #4 | 313 | 18.06.2024 |
| Filter | Filters applied: English, German. | 303 | 18.06.2024 |

(("nutritionist*"[Title/Abstract] OR "dietician*"[Title/Abstract] OR "dietitian*"[Title/Abstract] OR "nutrition* professional*"[Title/Abstract] OR "dietetic* student*"[Title/Abstract] OR "nutritionists"[MeSH Terms] OR "nutrition* student*"[Title/Abstract]) AND ("educat*"[Title/Abstract] OR "train*"[Title/Abstract] OR "curricul*"[Title/Abstract] OR "learn*"[Title/Abstract] OR "program*"[Title/Abstract] OR "teach*"[Title/Abstract] OR "pedagog*"[Title/Abstract] OR "course"[Title/Abstract] OR "educational status"[MeSH Terms] OR "education"[MeSH Terms]) AND ("sustainab*"[Title/Abstract] OR "environment*"[Title/Abstract] OR "climate change"[Title/Abstract] OR "planetary health"[Title/Abstract] OR "climate change"[MeSH Terms] OR "environment"[MeSH Terms]) AND ("competence*"[Title/Abstract] OR "literacy"[Title/Abstract] OR "capab*"[Title/Abstract] OR "knowledge"[Title/Abstract] OR "understand*"[Title/Abstract] OR "skill*"[Title/Abstract] OR "think*"[Title/Abstract])) AND (english[Filter] OR german[Filter])

**Scopus**

Table A2: Search in SCOPUS

| # | Search Query | Results | Date Run |
| --- | --- | --- | --- |
| #1 Population | TITLE-ABS-KEY ( dietician* ) OR TITLE-ABS-KEY ( dietitian* ) OR TITLE-ABS-KEY ( "nutrition* professionals" ) OR TITLE-ABS-KEY ( nutritionist* ) OR TITLE-ABS-KEY ( "nutrition* student*" ) OR TITLE-ABS-KEY ( "dietetic* student*" ) | [24,882](https://www.scopus.com/search/history/results.uri?origin=searchhistory&shid=10) | **18.06.24** |
| #2 Intervention – formal | TITLE-ABS-KEY ( educat* ) OR TITLE-ABS-KEY ( train* ) OR TITLE-ABS-KEY ( curricul* ) OR TITLE-ABS-KEY ( learn* ) OR TITLE-ABS-KEY ( program* ) OR TITLE-ABS-KEY ( teach* ) OR TITLE-ABS-KEY ( pedagog* ) OR TITLE-ABS-KEY ( course ) | [12,100,613](https://www.scopus.com/search/history/results.uri?origin=searchhistory&shid=11) | **18.06.24** |
| #3 Intervention – Content | TITLE-ABS-KEY ( sustainab* ) OR TITLE-ABS-KEY ( climate AND change ) OR TITLE-ABS-KEY ( environment* ) OR TITLE-ABS-KEY ( planetary AND health ) | [7,530,905](https://www.scopus.com/search/history/results.uri?origin=searchhistory&shid=12) | **18.06.24** |
| #4 Outcome | TITLE-ABS-KEY ( competenc* ) OR TITLE-ABS-KEY ( literacy ) OR TITLE-ABS-KEY ( capability ) OR TITLE-ABS-KEY ( knowledge ) OR TITLE-ABS-KEY ( understand* ) OR TITLE-ABS-KEY ( skills ) OR TITLE-ABS-KEY ( think* ) | [9,244,549](https://www.scopus.com/search/history/results.uri?origin=searchhistory&shid=13) | **18.06.24** |
| #5 | #1 AND #2 AND #3 AND #4 | 524 | **18.06.24** |
| Filter | Filters applied: English, German. | [484](https://www.scopus.com/search/history/results.uri?origin=searchhistory&shid=16) | **18.06.24** |

( TITLE-ABS-KEY ( competenc* ) OR TITLE-ABS-KEY ( literacy ) OR TITLE-ABS-KEY ( capability ) OR TITLE-ABS-KEY ( knowledge ) OR TITLE-ABS-KEY ( understand* ) OR TITLE-ABS-KEY ( skills ) OR TITLE-ABS-KEY ( think* ) ) AND ( TITLE-ABS-KEY ( sustainab* ) OR TITLE-ABS-KEY ( climate AND change ) OR TITLE-ABS-KEY ( environment* ) OR TITLE-ABS-KEY ( planetary AND health ) ) AND ( TITLE-ABS-KEY ( educat* ) OR TITLE-ABS-KEY ( train* ) OR TITLE-ABS-KEY ( curricul* ) OR TITLE-ABS-KEY ( learn* ) OR TITLE-ABS-KEY ( program* ) OR TITLE-ABS-KEY ( teach* ) OR TITLE-ABS-KEY ( pedagog* ) OR TITLE-ABS-KEY ( course ) ) AND ( TITLE-ABS-KEY ( dietician* ) OR TITLE-ABS-KEY ( dietitian* ) OR TITLE-ABS-KEY ( "nutrition* professionals" ) OR TITLE-ABS-KEY ( nutritionist* ) OR TITLE-ABS-KEY ( "nutrition* student*" ) OR TITLE-ABS-KEY ( "dietetic* student*" ) ) AND ( LIMIT-TO ( LANGUAGE , "English" ) OR LIMIT-TO ( LANGUAGE , "German" ) )

**Web of Science**

Table A3: Search in Web of Science

| # | Search Query | Results | Date Run |
| --- | --- | --- | --- |
| 1 (Population) | (((TS=(dieti?ian*)) OR TS=(nutrition* NEAR/5 professionals)) OR TS=(nutritionist*)) OR (TS=(nutrition* NEAR/5 student*)) OR (TS=(dietetic* NEAR/5 student*)) | 19,671 | 18.06.24 |
| 2 (Intervention – formal) | (((((((TS=(educat*)) OR TS=(train*)) OR TS=(curricul*)) OR TS=(learn*)) OR TS=(program*)) OR TS=(teach*)) OR TS=(pedagog*)) OR TS=(course*) | [7,557,710](https://www.webofscience.com/wos/woscc/summary/c801e8d6-fdc5-4d0c-8396-c56c00c88f56-f48ab109/relevance/1) | 18.06.24 |
| 3 (Intervention – Content) | (((TS=(sustainab*)) OR TS=(climate NEAR/5 change)) OR TS=(environment*)) OR TS=("planetary health") | [5,147,206](https://www.webofscience.com/wos/woscc/summary/01cc9587-ca33-4208-9058-81c48796edf6-f48aba2f/relevance/1) | 18.06.24 |
| 4 (Outcome) | (((((TS=(competence*)) OR TS=(literacy)) OR TS=(capability)) OR TS=(knowledge)) OR TS=(understand*)) OR TS=(skills) | [6,522,063](https://www.webofscience.com/wos/woscc/summary/1455a8b9-cc83-4418-91a3-cb14ace6fc2c-f48abf17/relevance/1) | 18.06.24 |
| 5 | #4 AND #3 AND #2 AND #1 | 515 | 18.06.24 |
| 6 (with filters) | #4 AND #3 AND #2 AND #1 and German or English (Languages) | 486 | 18.06.24 |

**Database: Web of Science Core Collection; (CPCI, SCI Expanded, SSCI und ESCI)**

**Filter: Language: German, English**

**ProQuest**

Table A4: Search in ProQuest

| # | Search Query | Results | Date Run |
| --- | --- | --- | --- |
| 1 (Population) | noft("nutritionist*" OR "dietician*" OR "dietitian*" OR "nutrition* professional*" OR "dietetic* student*" OR "nutrition* student*") | 10,967 | 18.06.24 |
| 2 (Intervention – formal) | noft("educat*" OR "train*" OR "curricul*" OR "learn*" OR "program*" OR "teach*" OR "pedagog*" OR "course" ) | 9,020,813 | 18.06.24 |
| 3 (Intervention – Content) | noft("sustainab*" OR "environment*" OR "climate change" OR "planetary health" ) | 2,634,305 | 18.06.24 |
| 4 (Outcome) | noft("competence*" OR "literacy" OR "capab*" OR "knowledge" OR "understand*" OR "skill*" OR "think*") | 4,691,358 | 18.06.24 |
| 5 | #4 AND #3 AND #2 AND #1 | 309 | 18.06.24 |
| 6 (with filters) | English language:  la.exact("ENG" NOT ("POR" OR "SPA")) | 305 | 18.06.24 |

All Databases included:

Databases: Acta Sanctorum, African Writers Series, Applied Social Sciences Index & Abstracts (ASSIA), ARTbibliographies Modern (ABM) for DFG, Bertolt Brechts Werke, British Periodicals, C19: The Nineteenth Century Index, Coronavirus Research Database, Digitale Bibliothek Deutscher Klassiker, Digitale Bibliothek Deutscher Klassiker, Digital National Security Archive, Early Modern Books, Ebook Central, ERIC, Gerritsen Women's History Collection of Aletta H. Jacobs, Goethes Werke, International Bibliography of Art (IBA), International Bibliography of the Social Sciences (IBSS), Linguistics and Language Behavior Abstracts (LLBA), Literature Online, PAIS Index, Periodicals Archive Online, Periodicals Index Online, ProQuest Historical Newspapers: Chicago Tribune, ProQuest Historical Newspapers: Los Angeles Times, ProQuest Historical Newspapers: The Boston Globe, ProQuest Historical Newspapers: The Guardian and The Observer, ProQuest Historical Newspapers: The Jewish Advocate, ProQuest Historical Newspapers: The Jewish Exponent, ProQuest Historical Newspapers: The New York Times, ProQuest Historical Newspapers: The Wall Street Journal, ProQuest Historical Newspapers: The Washington Post, PTSDpubs, Publicly Available Content Database, Schillers Werke, Social Services Abstracts, Sociological Abstracts, Sociological Abstracts, Sociology Database, Sports Medicine & Education Index, Sports Medicine & Education Index, Teatro Español del Siglo de Oro, The Annual Register: A Record of World Events, Worldwide Political Science Abstracts, Worldwide Political Science Abstracts

**Free search for grey literature**

**Google Scholar**

- | = OR; Space = AND; using quotation marks for phrases, no truncation
- **Retrieve the first 100 results each**
- Due to character limitations in Google Scholar's search bar (maximum 256 characters), multiple combinations of search terms were used to ensure comprehensive coverage.
- Screening of the first 100 results of each search, and only export “include” and “maybe” result to reference manager

Used combinations:

nutritionist|dietician AND education|learn AND sustainability|"climate change" AND competence|literacy

- Date run: 18.06.24 (included for screening n=8)

"nutrition professional"|"dietetic student"AND training|pedagogic AND "Planetary Health"|environment AND skills|knowledge

- Date run: 18.06.24 (included for screening n=4)

"nutrition student"|dietitians AND curriculum|program AND "sustainability"|environment AND capability|think

- Date run: 18.06.24 (included for screening n=1)

**Hand searching 🡪 citation tracking/ snowballing**

- Backward citation tracking: screening of the literature lists of included articles and non-primary articles

## Appendix 2: Quality appraisal of included studies

Table A5: Quality appraisal of included studies, using JBI checklists for qualitative, quasi-experimental and case report studies, and MMAT for mixed-methods and quantitative descriptive studies

| Study | JBI Checklist for Case Reports | | | | | | | | | |
| --- | --- | --- | --- | --- | --- | --- | --- | --- | --- | --- |
|  | C1 | C2 | C3 | C4 | C5 | C6 | C7 | C8 |  |  |
| Brekken, C. A. et al. (2018) | N | Y | N | N | Y | N | Y | Y |  |  |
| Bustamante, M. A. et al. (2016) | N | Y | N | Y | Y | Y | N | Y |  |  |
| Fox, A. (2019) | N | Y | N | Y | Y | Y | Y | Y |  |  |
| Matthews, J. (2013) | N | Y | N | Y | Y | Y | Y | Y |  |  |
| Pontikis, K. et al. (2011) | N | Y | N | N | Y | N | N | Y |  |  |
|  | **JBI Checklist for Qualitative Research** | | | | | | | | | |
|  | C1 | C2 | C3 | C4 | C5 | C6 | C7 | C8 | C9 | C10 |
| Carino, S. et al. (2019) | N | Y | Y | Y | Y | N | N | N/A | Y | Y |
| Maher, J. and Burkhart, S. J. (2017) | N | Y | Y | Y | Y | Y | N | Y | Y | Y |
| Miller, M. et al. (2024) | N | Y | Y | Y | Y | Y | N | Y | Y | Y |
| Wadsworth, L. et al. (2012) | Y | Y | Y | Y | Y | Y | N | Y | Y | Y |
| Pabani, N. et al. (2020) | Y | Y | Y | Y | Y | N | Y | Y | Y | Y |
| Ruhl, J. and Lordly, D. (2021) | N | Y | Y | Y | Y | N | N | Y | Y | Y |
|  | **JBI Checklist for Quasi-Experimental Studies** | | | | | | | | | |
|  | C1 | C2 | C3 | C4 | C5 | C6 | C7 | C8 | C9 |  |
| Spiker, M. et al. (2021)  Outcome: ability to perform the learning outcomes | Y | N | Y | Y | Y | Y | N | Y | Y |  |
| Shafto, K. et al. (2023)  Outcome 1: attitudes and knowledge | Y | N | Y | Y | Y | Y | Y | N | Y |  |
| Shafto, K. et al. (2023)  Outcome 2: personally meaningful insights or topics | Y | N | Y | Y | N | Y | N | N | N/A |  |
| Navarro, V. et al. (2019)  Outcome 1: competence knowledge | Y | Y | Y | Y | Y | Y | N | N | Y |  |
| Navarro, V. et al. (2019)  Outcome 2: sustainability knowledge | Y | Y | Y | Y | Y | Y | N | N | Y |  |
| Navarro, V. et al. (2019)  Outcome 3: sustainable intentional behaviour | Y | Y | Y | Y | Y | Y | N | N | Y |  |
| Innes, S. et al. (2018) Outcome: environmental literacy | Y | N | Y | Y | Y | Y | N | Y | Y |  |
| Knobloch, C. et al. (2019)  Outcome 1: perceptions of learning experiences and outcomes | Y | N | Y | Y | Y | Y | Y | N | Y |  |
| Knobloch, C. et al. (2019)  Outcome 2: perceptions of the seminar | Y | N | Y | Y | N | Y | N | N/A | N/A |  |
| Knobloch, C. et al. (2019)  Outcome 3: benefits, barriers, and motivational factors regarding faculty collaboration | Y | N | Y | Y | Y | Y | Y | Y | N |  |
|  | **MMAT for mixed methods studies** | | | | | | | | | |
|  | S1 | S2 | 5.1 | 5.2 | 5.3 | 5.4 | 5.5 |  |  |  |
| Holik, M. et al. (2021) | Y | Y | Y | Y | Y | N | Y |  |  |  |
| Meyer, N. et al. (2021) | Y | Y | Y | Y | Y | N | Y |  |  |  |
|  | **MMAT for quantitative studies** | | | | | | | | | |
|  | S1 | S2 | 4.1 | 4.2 | 4.3 | 4.4 | 4.5 |  |  |  |
| Hege, A. et al. (2021) | Y | Y | Y | N | Y | N | Y |  |  |  |

Abbreviations: JBI Johanna Briggs Institute; MMAT Mixed Methods Appraisal Tool; C criteria; Y Yes; N No; N/A Not applicable

**Criteria of JBI Checklist for Case Reports**

C1 Were student’s demographic characteristics clearly described?

C2 Was the student’s prior educational history clearly described (and presented as a timeline)?

C3 Was the initial learning status of the students before the intervention clearly described?

C4 Were evaluation methods and the results clearly described?

C5 Was the intervention(s) or teaching method(s) clearly described?

C6 Was the students’ learning progress or post-intervention condition/effect clearly described?

C7 Were adverse events or unanticipated events identified and described?

C8 Does the case report provide takeaway lessons?

**Criteria of JBI Checklist for Qualitative Research**

C1 Is there congruity between the stated philosophical perspective and the research methodology?

C2 Is there congruity between the research methodology and the research question or objectives?

C3 Is there congruity between the research methodology and the methods used to collect data?

C4 Is there congruity between the research methodology and the representation and analysis of data?

C5 Is there congruity between the research methodology and the interpretation of results?

C6 Is there a statement locating the researcher culturally or theoretically?

C7 Is the influence of the researcher on the research, and vice-versa, addressed?

C8 Are participants, and their voices, adequately represented?

C9 Is the research ethical according to current criteria or, for recent studies, and is there evidence of ethical approval by an appropriate body?

C10 Do the conclusions drawn in the research report flow from the analysis, or interpretation, of the data?

**Criteria of JBI Checklist for Quasi-Experimental Studies**

C1 Temporal precedence

C2 Selection and allocation

C3 Confounding factors

C4 Administration of intervention/exposure

C5 Assessment of the outcome

C6 Detection of the outcome

C7 Measurement of the outcome

C8 Participant retention

C9 Statistical conclusion validity

**Criteria of MMAT**

S1. Are there clear research questions?

S2. Do the collected data allow to address the research questions?

4.1. Is the sampling strategy relevant to address the research question?

4.2. Is the sample representative of the target population?

4.3. Are the measurements appropriate?

4.4. Is the risk of nonresponse bias low?

4.5. Is the statistical analysis appropriate to answer the research question?

5.1. Is there an adequate rationale for using a mixed methods design to address the research question?

5.2. Are the different components of the study effectively integrated to answer the research question?

5.3. Are the outputs of the integration of qualitative and quantitative components adequately interpreted?

5.4. Are divergences and inconsistencies between quantitative and qualitative results adequately addressed?

5.5. Do the different components of the study adhere to the quality criteria of each tradition of the methods involved?
